# Phenolics as Active Ingredients in Skincare Products: A Myth or Reality?

**DOI:** 10.3390/molecules30071423

**Published:** 2025-03-23

**Authors:** Ana Jesus, Smeera Ratanji, Honorina Cidade, Emília Sousa, Maria T. Cruz, Rita Oliveira, Isabel F. Almeida

**Affiliations:** 1 Associate Laboratory i4HB—Institute for Health and Bioeconomy, Faculty of Pharmacy, University of Porto, 4050-313 Porto, Portugal; anaaimjesus@gmail.com (A.J.); smeeraratanji@gmail.com (S.R.); ritao@ufp.pt (R.O.); ifalmeida@ff.up.pt (I.F.A.); 2 UCIBIO—Applied Molecular Biosciences Unit, Department of Drug Sciences, Faculty of Pharmacy, University of Porto, 4050-313 Porto, Portugal; 3 Laboratory of Organic and Pharmaceutical Chemistry, Department of Chemical Sciences, Faculty of Pharmacy, University of Porto, 4050-313 Porto, Portugal; 4 CIIMAR—Interdisciplinary Centre of Marine and Environmental Research, 4450-208 Matosinhos, Portugal; 5 UNIPRO—Oral Pathology and Rehabilitation Research Unit, University Institute of Health Sciences (CESPU), 4585-116 Gandra, Portugal; 6 Faculty of Pharmacy, University of Coimbra, 3004-531 Coimbra, Portugal; trosete@ff.uc.pt; 7 CNC—Center for Neuroscience and Cell Biology, 3004-504 Coimbra, Portugal; 8 FP-BHS—Biomedical and Health Sciences Research Unit, FFP-I3ID Faculty of Health Sciences, University of Fernando Pessoa, 4200-150 Porto, Portugal; 9 RISE—Health, Faculty of Health Sciences, Fernando Pessoa University, Fernando Pessoa Teaching and Culture Foundation, Rua Carlos da Maia 296, 4200-150 Porto, Portugal

**Keywords:** phenolic compounds, skin aging, mechanism of action, suncare cosmetic products, anti-aging cosmetic products

## Abstract

Phenolic compounds, with their diverse biological activities, are widely explored as cosmetic ingredients with photoprotective, antioxidant, anti-inflammatory, and anti-hyperpigmentation properties, offering a multitargeted approach to combat photo-induced skin aging. The study analyzed 1299 cosmetic products from 2021 to 2024 to understand the market impact of phenolic compounds and their mechanism of action against photo-induced skin damage. A total of 28 active phenolic compounds were identified and the prevalence of phenolics was 13.2% in anti-aging products, 5.2% in sunscreens and 4.8% in aftersun products. Bakuchiol and polyphenols, such as resveratrol, chrysin, and hesperidin methyl chalcone, were found in anti-aging products. Sunscreens and aftersun products were counted with ferulic and caffeic acids, and salicylic acid, respectively. Antioxidant activity was found to be the primary mechanism of action of phenolic compounds by scavenging reactive species, thus mitigating oxidative stress. Ferulic and caffeic acids, chrysin, and glucosylrutin can also absorb UV radiation, acting preventively against solar-induced skin damage. This study provides insights into the limited use of phenolic compounds in commercial cosmetics, despite their diverse biological activities, and suggests potential barriers to wider use in skin and sun care products.

## 1. Introduction

Phenolic compounds, a broad category of naturally derived organic molecules commonly found in botanical species [1], have become the focus of innovation in the cosmetic industry. Their distinctive chemical structure includes at least one hydroxy group connected to an aromatic ring, empowering them with several biological functions [2]. Phenolic compounds come in many different structural forms and can be divided using different classification systems, based on the number of carbons in the molecule, into simple phenolics and polyphenols [2,3]. Generally, both simple phenolics and polyphenols have been extensively documented for their potent ability to scavenge various reactive oxygen species (ROS), including superoxide anion radicals (O_2_^∙−^), hydroxy radicals (HO^∙^), hydrogen peroxide (H_2_O_2_), and singlet oxygen (^1^O_2_) [4]. Phenolic acids, including hydroxybenzoic (C_6_-C_1_ general chemical structure) and hydroxycinnamic acids (C_6_-C_3_ general chemical structure), have been also reported for their anti-inflammatory and anti-aging activities [2,3,5]. Among polyphenolic compounds, flavonoids (C_6_-C_3_-C_6_ general chemical structure) stand out as one of the most extensive and well-researched chemical classes [3,6,7]. Flavonoids form a group of structurally diverse natural products, with a C_6_-C_3_-C_6_ scaffold, which includes anthocyanidins, aurones, chalcones, flavones, flavanones, flavanols, and flavonols, and are present in many foods and plants, including fruits, vegetables, tea, and wine [1,4]. Flavonoids are well known for their antioxidant, anti-inflammatory, and anti-pigmentation properties, as well as their ability to absorb solar radiation [4].

Phenolic compounds are naturally occurring bioactive secondary metabolites, and their diverse structural forms are mainly dependent on their biosynthesis. The biosynthetic production of these compounds occurs through two main metabolic pathways: the shikimate and acetate–malonate pathways [4]. The shikimate pathway is based on shikimic acid, which is the primary precursor in the biosynthesis of aromatic amino acids. Additional transformations can occur and shikimic acid can give rise to hydroxybenzoic acids, and consequently to hydroxycinnamic acids [4]. The acetate–malonate pathway, based on malonyl coenzyme A (CoA) units, is often integrated with the phenylpropanoid pathway, resulting in the production of flavonoids. In the flavonoid’s structure, ring B is derived from the shikimate pathway via hydroxycinnamic acid, while ring A is formed through the acetate–malonate pathway [8]. The process involves a reaction between three malonyl CoA units (acetate–malonate pathway) and one coumaroyl CoA unit (shikimate pathway), forming a thioester-linked tetraketide intermediate under the catalytic action of the enzyme chalcone synthase. This intermediate undergoes enolization, leading to the formation of the chalcone nucleus, which serves as the precursor for various flavonoid subclasses [4,8].

Since 2020, over 40 research articles have been identified using the keywords “flavonoids” and “skin care,” with the number doubling when searching for “flavonoids” and “skin aging”. Scientific research has highlighted their advantages for skin care, especially their capacity to alleviate inflammation, fight oxidative stress, and shield the skin from UV radiation [9,10]. Despite their relative abundance in nature when compared with flavonoids, stilbenes [11,12] and xanthones [13,14] have demonstrated a substantial ability to combat oxidative stress and contribute to the regulation of melanin synthesis. Exposure to solar radiation triggers a set of detrimental effects on the skin, including the production of ROS, exacerbating mitochondrial and cellular oxidative stress, biomolecule damage, and an increase in inflammatory response [15,16]. Phenolic compounds have shown a promising profile in fighting these main events, by effectively neutralizing ROS, shielding DNA from direct and oxidative damage, minimizing inflammation, preventing collagen degradation through the inhibition of proteolytic enzymes, and regulating melanin synthesis [17]. These actions can presumably make phenolic compounds valuable ingredients in skincare products designed to protect against sun damage and maintain skin health.

Given the importance of phenolic compounds in fighting the negative effects of solar radiation, it is essential to identify and comprehend the specific mechanism of action of phenolic compounds found in cosmetic product. This article sets out to provide a thorough assessment of the phenolic compounds present in different categories of skincare products, particularly suncare and anti-aging products, and examines the scientific evidence supporting their effectiveness, shedding light on the mechanism of action of phenolic compounds in skincare formulations.

## 2. Results and Discussion

### 2.1. Overview of the Presence of Phenolic Compounds in Each Category of Products

**Figure 1:**
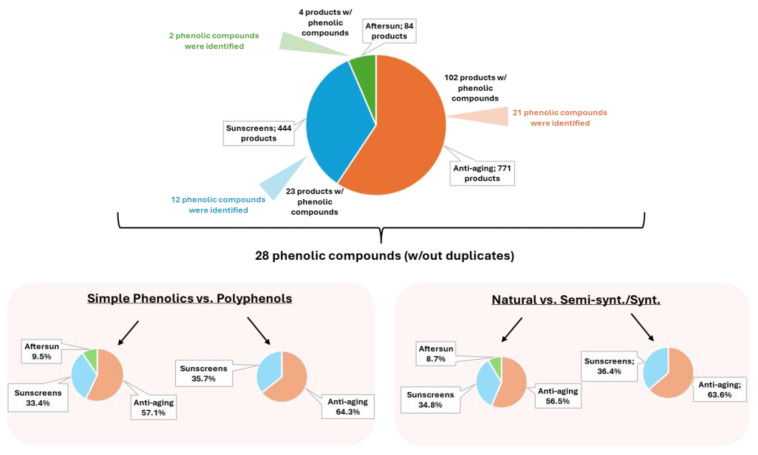
Analysis of the presence of phenolic ingredients in each category of products, according to their chemical classification and source. Semi-synt./synt.: semi-synthetic/synthetic compounds, w/: with, w/out: without.

A total of 1299 products from all of the categories of products studied (anti-aging, sunscreen, and aftersun) allowed us to identify 137 international brands, 27 of which were exclusively marketed by Portuguese pharmacies. From all of the products, the prevalence of phenolic compounds varied between 0.2% and 15%. Among all of the 771 anti-aging products, 102 products presented phenolic compounds in their constitution (13.2%), followed by sunscreen products (5.2%; 23 products of 444), and aftersun products (4.8%; four products of 84) (Figure 1). A total of 28 phenolic compounds was identified in all the three categories of cosmetic products analyzed. From all these categories, 16 simple phenolic compounds were found in the three categories of products (anti-aging: 57.1%, 12 compounds; sunscreens: 33.4%, 7 compounds; and aftersun: 9.5%, 2 compounds), contrary to what was observed for the 12 polyphenols that were only detected in anti-aging (64.3%, nine polyphenols), and in sunscreen (35.7%, five polyphenols) products. A total of 13 natural phenolic ingredients were found in anti-aging products (56.5%), followed by 8 ingredients in sunscreens (34.8%) and 2 ingredients in aftersun products (8.7%) from natural sources. On the other hand, the 11 semi-synthetic/synthetic phenolic compounds demonstrated a similar distribution to the polyphenols, being present in only two categories of products, seven ingredients in anti-aging products (63.6%), and four in sunscreens (36.4%).

### 2.2. Usage Frequency of Phenolic Compounds in Cosmetic Products

#### 2.2.1. Anti-Aging Products

**Figure 2:**
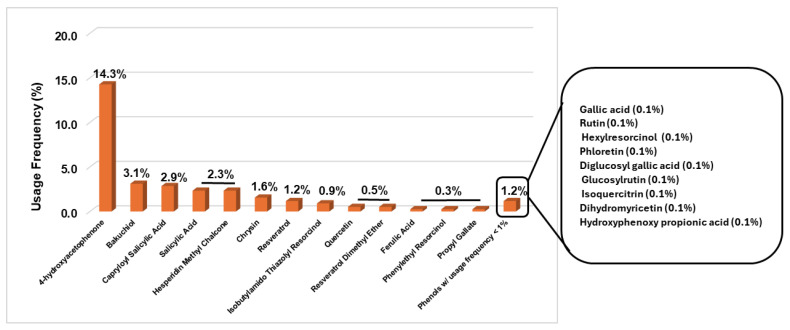
Phenolic compounds found in anti-aging products marketed in 2024.

In the analysis of anti-aging products, a total of 771 formulations were examined, revealing the presence of 22 phenolic compounds. Hydroxyacetophenone (4-hydroxyacetophenone) was the most frequently used, appearing in 110 products (14.3%). Six other phenolic compounds were identified with usage frequencies between 1% and 10%, including three polyphenols—hesperidin methyl chalcone (2.3%), chrysin (1.6%), and resveratrol (1.2%)—and three simple phenolics—bakuchiol (3.1%), capryloyl salicylic acid (2.9%), and salicylic acid (2.3%). Nine ingredients found in anti-aging products with usage frequencies below 1% accounted for 1.2% of the total phenolic compounds identified, displaying an equitable distribution between simple phenols and polyphenols (Figure 2).

#### 2.2.2. Sunscreens Products

**Figure 3:**
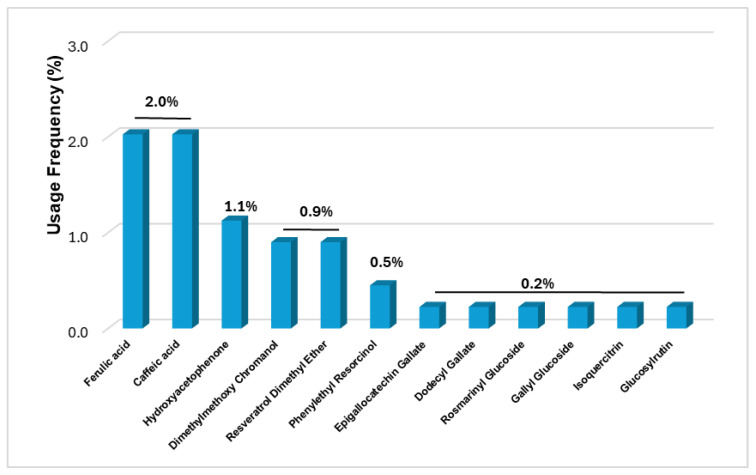
Usage frequency of phenolic compounds found in sunscreen products marketed in 2021.

In the pool of 444 collected sunscreen products, a total of 12 phenolic compounds were pinpointed (Figure 3). The most used phenolic compounds were from naturally derived sources, ferulic and caffeic acid (2.0% each, in nine products), and hydroxyacetophenone, with a usage frequency of 1.1% (five products). The remaining phenolic compounds were found in four or less products, with usage frequencies inferior to 1% (five naturally derived—epigallocatechin gallate, rosmarinyl glucoside, gallyl glucoside, isoquercitrin, and glucosylrutin—and four from semi-synthetic/synthetic sources—dimethylmethoxy chromanol, resveratrol dimethyl ether, phenylethyl resorcinol, and dodecyl gallate).

#### 2.2.3. Aftersun Products

**Figure 4:**
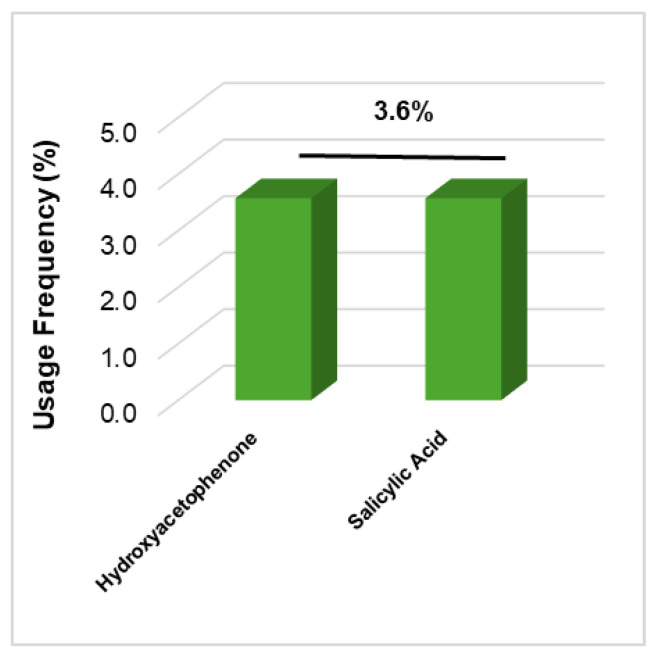
Usage frequency of phenolic compounds found in aftersun products marketed in 2023 and 2024.

Among the 84 aftersun products analyzed, only two phenolic compounds were identified (Figure 4). Only hydroxyacetophenone and salicylic acid were identified as active phenolic compounds in aftersun products (three products each), with their usage frequency being 3.6% each. All of the phenolic compounds identified in this class of cosmetic products belong to the simple phenolics class and are from natural sources.

#### 2.2.4. Other Phenolics

Seven phenolic compounds with antioxidant properties were identified; however, their primary role in cosmetic formulations is as stabilizers, preservatives, fragrances, or colorants. Nonetheless, these compounds remain of interest and could offer alternatives to currently used ingredients with similar functions in cosmetic formulations. Tocopherol was the phenolic compound with the highest usage frequency (≥50%) and was present in all three categories of products, demonstrating its versatility. Tocopherol, with its chromanol ring structure, exhibits potent antioxidant activity and plays a regulatory role in signaling pathways linked to cellular apoptosis and proliferation [18]. Additionally, it mitigates inflammatory responses by reducing prostaglandin levels and pro-inflammatory cytokines, such as interleukin-1β and interleukin-8 [19,20,21]. Tocopherol acts as a free radical scavenger, protecting skin cells from oxidative stress [22,23], neutralizing lipid peroxy radicals [24], and combating DNA oxidative damage by decreasing the levels of 8-hydroxyguanosin, and 8-hydroxy-2-deoxyguanosine [25,26].

Butylated hydroxytoluene (80 anti-aging products, 10.4%; 84 sunscreen products, 18.9%; and 6 aftersun products, 7.1%), butylated hydroxyanisole (10 anti-aging products, 1.3%), and pentaerythrityl tetra-di-*t*-butyl hydroxyhydrocinnamate (55 anti-aging products, 7.1%; and 2 aftersun product, 2.4%) are synthetic antioxidants with various applications in the food, pharmaceutical, and cosmetics fields [27,28]. Butylated hydroxytoluene and butylated hydroxyanisole also act as stabilizers to prevent product deterioration [28,29].

Coumarin was found in 52 anti-aging (6.7%) and in 6 aftersun (7.1%) products; however, its main role in cosmetic formulations is as a fragrance and colorant due to its sweet, vanilla-like scent and yellow color, respectively [30]. Several studies have explored the potential of *2H*-1-benzopyran-2-one derivatives as UV absorbers [31,32], wound-healing agents [33], anti-inflammatory actives [34], inhibitors of metalloproteinases [35,36], and anti-melanogenic compounds [37].

Bis-ethylhexyl hydroxydimethoxy benzylmalonate (two sunscreen products, 0.5%) has antioxidant properties and photostabilizer characteristics, namely for photo-unstable UV filters [38,39]. Diethylhexyl syringylidenemalonate (14 anti-aging products, 1.8%) is also a phenolic compound that acts as an effective photostabilizer by helping to maintain the original color of products, preventing color fading, and enhancing the stability of photo-unstable UV filters [40,41,42].

### 2.3. Scientific Evidence Supporting the Effectiveness of the Phenolic Compounds

The phenolic compounds with usage frequencies above 1% or present in at least two product categories will be discussed in terms of their effectiveness. Table 1 reviews the occurrence of all the phenolic compounds that will be further described.

A critical scientific discussion was conducted on the scientific evidence of the 13 phenolic compounds listed in Table 1 in combating skin aging through various mechanisms. These include absorbing radiation (sunscreens), preventing oxidative stress by neutralizing reactive oxygen species (ROS) (sunscreens, aftersun, and anti-aging products), reducing inflammatory processes (aftersun and anti-aging products), interrupting senescence (a state of terminal cellular growth arrest) (anti-aging products), and regulating melanin production (sunscreens and anti-aging products).

#### 2.3.1. Simple Phenols

##### Hydroxyacetophenone

**Figure 5:**
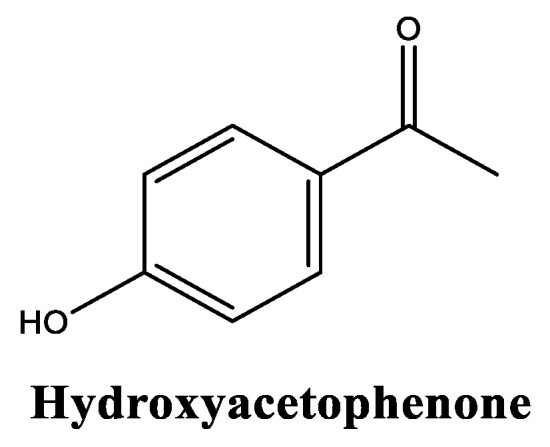
Chemical structure of hydroxyacetophenone.

Hydroxyacetophenone (Figure 5), chemically denominated as 4-hydroxyacetophenone, is naturally found in some plants, specifically in Norwegian spruce (*Picea abies*) needles at concentrations between 0.4% and 2.8%, and it can also be extracted from cloudberries (*Rubus chamaemorus*) [43,44]. To be used in the cosmetic industry, hydroxyacetophenone is primarily produced in a more efficient, synthetic manner [45]. It has been deemed safe for use in cosmetics at typical concentrations (up to 5% for leave-on products), with no significant reports of dermal irritation or sensitization, and presents a solubility in water of 10 g/L [45]. Hydroxyacetophenone is a versatile ingredient used in cosmetics and skincare products for its multiple beneficial properties, including as a potent free radical scavenger [46], by protecting skin cells from oxidative stress and for lipid peroxidation [47]. This phenolic also has anti-inflammatory activity [48], modulating cyclooxygenase 2 enzyme activity, helping to reduce skin inflammation and diminishing irritation reactions [49]. Hydroxyacetophenone also acts as a skin-conditioning agent, improving the overall appearance and smoothness of the skin [45]. Although there is no claim that hydroxyacetophenone possesses preservative properties on its own, it has been shown to enhance the effectiveness of other preservatives in formulations [50].

##### Bakuchiol

**Figure 6:**
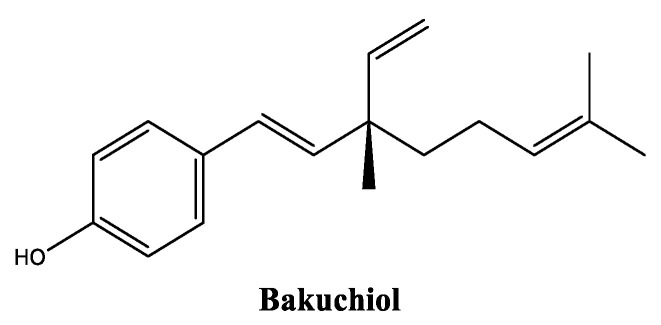
Chemical structure of bakuchiol.

Bakuchiol (Figure 6) is a naturally derived monoterpene from the seeds of the plant *Psoralea corylifolia*, also known as babchi, widely used in Asian countries for traditional medicine treatments [51]. Bakuchiol is produced in nature via a mixed biosynthetic route involving the mixture of isoprene-based and amino acid-based building blocks, the aromatic ring of bakuchiol in particular is formed from a shikimate pathway and the monoterpene side chain comes from the mevalonate pathway [52]. This has gained popularity in skincare due to its adequate profile for sensitive skin, as it has already been reported to be a gentler alternative to retinol, offering similar benefits with potentially fewer side effects [53,54]. Bakuchiol is generally well tolerated, with a lower risk of irritation compared with retinoids, and it is typically used in oil-based formulations, suggesting good solubility in lipophilic liquids [55]. Bakuchiol has been reported for its antioxidant [56], anti-inflammatory [57,58], and anti-aging properties [51,59], namely by in vitro neutralizing reactive species [56], suppressing the in vitro and in vivo production of prostaglandin E2 and interleukin-6 [57], inhibiting the in vitro expression of the inducible form of nitric oxide synthase and cyclooxygenase-2 [58], in vivo reducing fine lines and wrinkles [59,60], as well as improving skin elasticity, stimulating collagen production, and, in vitro, accelerating skin turnover [59]. A good tolerability was observed in clinical studies by significantly decreased wrinkle surface area and hyperpigmentation [55,60]. Through these two possible mechanisms, bakuchiol regulates the synthesis of melanin, when both are overexpressed. Moreover, bakuchiol thereby acts against the decline of fibroblast growth factor levels that occur during aging, which is common in senescent cells [54]. This highlights the multifunctionality of the ingredient and its potential to target multiple pathways in the fight against skin aging.

##### Salicylic Acid and Capryloyl Salicylic Acid

**Figure 7:**
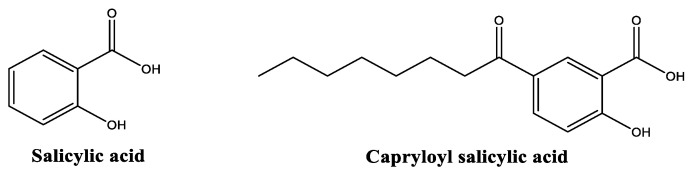
Chemical structure of salicylic acid and capryloyl salicylic acid.

Salicylic acid (Figure 7) is a β-hydroxy acid widely used in skincare for its numerous benefits, namely in acne management, exfoliation, and oil regulation [61,62]. It also acts as an antioxidant [63,64] and has the ability to decrease skin hyperpigmentation through the inhibition of melanosome transport [65]. The cleansing and exfoliation properties of salicylic acid indirectly contribute to the reduction of skin inflammation [61]. Salicylic acid is typically found in concentrations between 0.5% and 2% in leave-on products, and the maximum concentration could be increased to 30% in rinse-off products [66]. Salicylic acid has a more stable profile at acidic pH values and a poor water solubility, probably limiting the skin penetration [66]. Capryloyl salicylic acid (Figure 7) is a derivative of salicylic acid and thus also possesses some of the properties of the parent compound. Its exfoliation and cleansing properties [62] are considered better than those of salicylic acid due to its aliphatic chain, which can result in a higher affinity for the lipophilic components of the skin barrier [67]. It is also better tolerated by sensitive skin types and offers improved stability in cosmetic formulations [66]. Capryloyl salicylic acid maintains the same ability to promote cellular turnover and improve skin texture, regulate sebum production, and reduce inflammatory clinical signs, including redness and irritation [68], as its parent compound. Capryloyl salicylic acid demonstrates a superior water solubility, pH stability, and gentler and less irritant characteristics for the skin when compared with the salicylic acid [66].

A split-face clinical trial examined 50 women aged between 35 and 60 with signs of facial hyperpigmentation and wrinkles [68]. Participants received a formulation containing capryloyl salicylic acid (5–10%) on one face side, and glycolic acid (20–50%) on the other, enabling direct treatment comparison within the same individual [68]. A 12-week treatment period demonstrated that capryloyl salicylic acid at 5–10% was comparable in safety and effectiveness to glycolic acid at 20–50% in the same formulation and time. Both treatments showed similar results in diminishing hyperpigmentation and skin wrinkles [68].

##### Ferulic and Caffeic Acids

**Figure 8:**
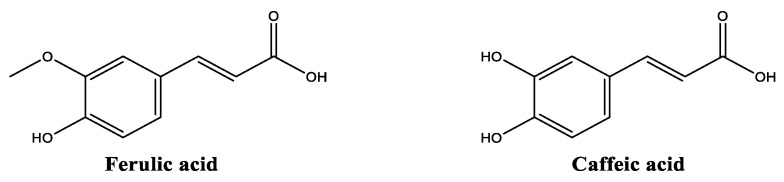
Chemical structure of ferulic and caffeic acids.

Ferulic and caffeic acids (Figure 8) are both found in numerous plant sources, grains (rice, wheat, and oats), fruits (pineapple, banana, orange, and grapes), vegetables (eggplant, spinach, broccoli, and tomato), and other foods (coffee beans, artichoke, and peanuts) [69]. Both hydroxycinnamic acids are more prone to be soluble in lipophilic components, with caffeic acid moderately soluble in water and ethanol due to the presence of two hydroxy groups in its structure, compared with the one hydroxy group of the ferulic acid. These are considered sensitive to light, heat and oxygen, susceptible to degradation, and possess a more stable profile in acidic pH values, being their encapsulation the most employed strategy to improve their stability [70]. Ferulic acid is a hydroxycinnamic acid and a well-known antioxidant compound, with the ability to neutralize reactive species and protect skin from extrinsic factors, including solar radiation and pollution [71,72]. Due to its antioxidant properties, it can act synergistically with other antioxidants, namely ascorbic acid and retinol [71,72,73], helping not only to protect from externally triggered ROS but also to contribute to the stability of the formulation [70]. Ferulic acid also possesses anti-aging benefits [70], by improving the skin’s overall appearance, firmness, and elasticity, and by activating collagen and elastin production [74,75]. Ferulic acid effectively reduces inflammation and oxidative stress in vitro, through the inhibition of the NADPH oxidase, and via the downregulation of the mitogen active protein kinase (MAPK) and AKT pathways [75]. Ferulic acid has also been reported to feature some photoprotective characteristics, specifically by boosting UV radiation absorption when combined with other photoprotective ingredients [72]. Ferulic acid was also found to protect skin from UV-induced structural protein damage, avoiding DNA damage and inhibiting in vivo the UVB activation of metalloproteinases-2 and -9 responsible for extracellular fiber degradation [70,76].

As with ferulic acid, caffeic acid also exerts a relevant reactive species scavenger activity, protecting skin from oxidative stress [77], as well as offering anti-inflammatory [78,79], and anti-aging properties [77]. Moreover, caffeic acid has been reported for its antimicrobial activity [80], potentially helping in cosmetic product preservation and skin healing. Additionally, both ferulic and caffeic acids exhibit effective action towards melanin synthesis through different mechanisms of actions in B16 melanoma cells [81]. Ferulic acid reduces tyrosinase enzyme activity by directly binding to its active site, in contrast to caffeic acid, which interacts with α-melanocyte-stimulating hormone [82] but not with the enzyme’s active site [81]. Both ferulic acid and caffeic acid inhibit casein kinase 2-induced phosphorylation of tyrosinase in a dose-dependent manner in vitro [81].

##### Phenylethyl Resorcinol

**Figure 9:**
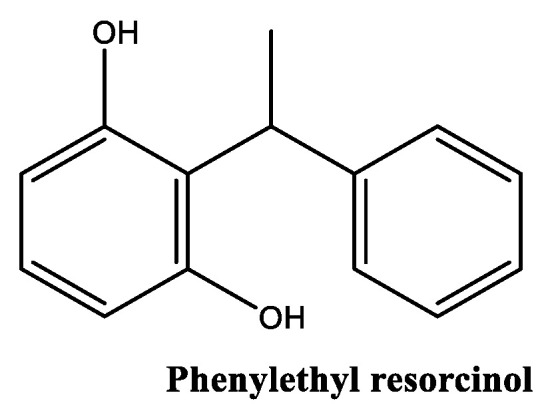
Chemical structure of phenylethyl resorcinol.

Phenylethyl resorcinol (Figure 9) is a synthetic racemic mixture with a lipophilic characteristics, derived from resorcinol, a naturally occurring phenolic [83] and inspired by natural compounds found in Scotch pine bark [84]. In cosmetic products, phenylethyl resorcinol is generally considered safe for use at concentrations between 0.1% and 1.0%, and has a better stability profile between pH values of 4 and 6 [85]. This derivative of resorcinol is known for its skin-lightening properties, due to its ability to act as a competitive inhibitor of tyrosinase through the activation of the p44/42 mitogen-activated protein kinase signaling pathway [86], reducing the production of melanin in the skin. Its potency surpasses the well-known inhibitor of tyrosinase, kojic acid [87]. According to clinical studies, phenylethyl resorcinol at a concentration of 0.5% in cosmetic products exhibits a superior effective response, more effectively lightening skin than 1% concentration of kojic acid [85,88]. Moreover, phenylethyl resorcinol outperforms the antioxidant activity (scavenger of the 2,2-diphenyl-1-picrylhydrazyl (DPPH) radical) of tocopherol, ascorbic acid and butylated hydroxytoluene [84,89,90]. This derivative also displays some drawbacks, namely poor photo-stability and low water solubility, which limits its use in hydrophilic formulations [91]. Liu and colleagues have studied phenylethyl resorcinol formulation using in vitro and clinical studies. Two studies examined UV-induced pigmentation and skin hyperpigmentation in a total of 86 participants [89]. The research demonstrated significant improvements in pigmentation reduction and skin radiance. Key findings include notable skin brightening effects, improvements that were maintained even after product discontinuation, and effective treatment for solar lentigo and post-acne hyperpigmentation [89]. As far as we know, phenylethyl resorcinol lacks direct scientific evidence for anti-senescence and anti-inflammatory activities. However, potent antioxidant and anti-hyperpigmentation properties are relevant mechanisms by which to combat solar-induced skin aging.

#### 2.3.2. Polyphenols

##### Hesperidin Methyl Chalcone

**Figure 10:**
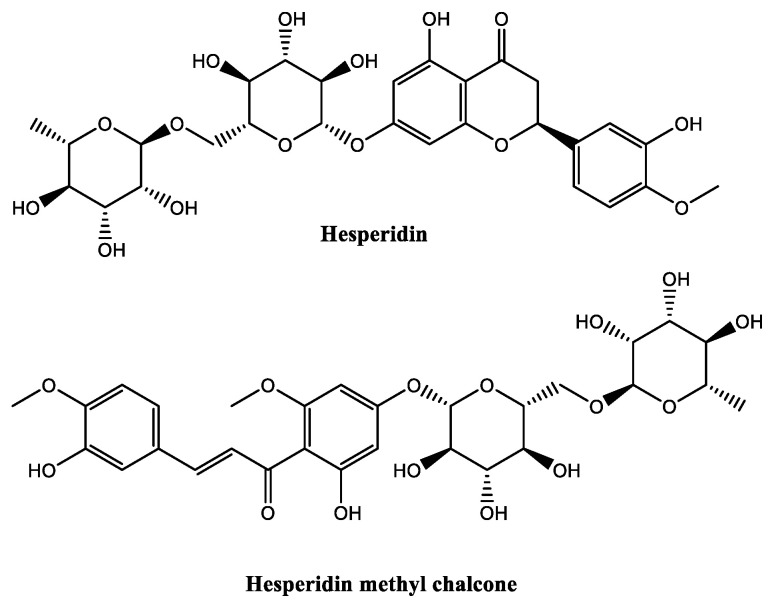
Chemical structure of hesperidin methyl chalcone.

Hesperidin methyl chalcone (Figure 10) is a semi-synthetic derivative from hesperidin that belongs to the flavonoid family, commonly found in citrus fruits, specifically in the peel of oranges [7]. Hesperidin (Figure 10) is first extracted from natural sources and is subsequently subjected to a methylation reaction under alkaline conditions, resulting in the formation of hesperidin methyl chalcone [92]. Hesperidin methyl chalcone is used in concentrations typically ranging from 0.05 to 2%, and an improved solubility (water, and alcohol solvents) and stability (pH = 2–7.4, and at temperatures <50 °C) when compared with hesperidin [93]. This derivative possesses radical quenching properties [94], and reduces skin inflammation, through the inhibition of the expression of metalloproteinases and interleukins [94,95]. This also promotes skin barrier repair by increasing filaggrin expression, as lower levels of filaggrin can lead to disorganization and alteration in the composition of ceramides within the skin barrier [93,96,97]. Hesperidin methyl chalcone possesses better stability and compatibility with other ingredients when compared with its parent compound hesperidin [98]. Hesperidin methyl chalcone demonstrated significant antioxidant and anti-inflammatory properties in both in vitro and in vivo studies [96]. This effectiveness of this hesperidin derivative was evaluated using a mouse model of UVB-induced skin oxidative stress and inflammation [96]. In in vitro studies, hesperidin methyl chalcone exhibited Fe^2+^ chelating effects, neutralized hydroxy reactive species, and inhibited lipid peroxidation, while in vivo studies on hairless mice showed that the topical application of this ingredient inhibited UVB-induced skin oedema, maintained glutathione levels by preserving glutathione peroxidase-1 and glutathione reductase enzyme expression, prevented downregulation of nuclear factor erythroid 2-related factor 2, and inhibited pro-inflammatory cytokines (interleukin-1β, and -6) [96].

##### Chrysin

**Figure 11:**
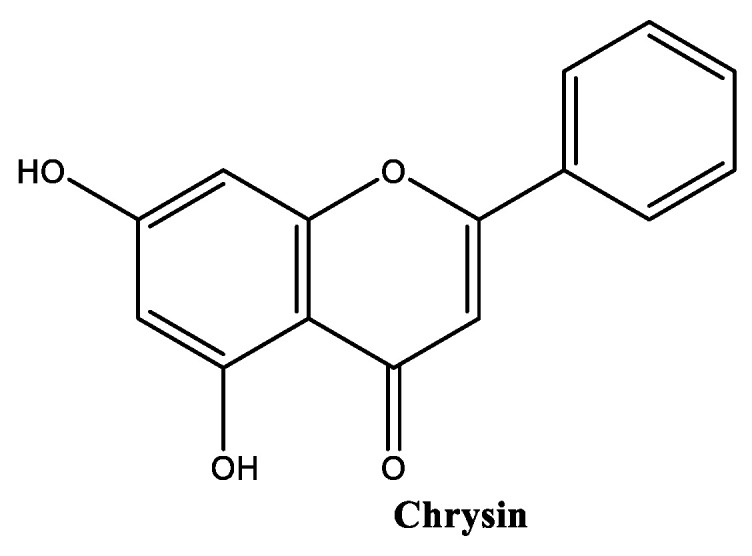
Chemical structure of chrysin.

Chrysin (Figure 11), also known as 5,7-dihydroxyflavone, is a naturally occurring flavonoid found in various plant sources (*Passiflora caerulea*, *Passiflora incarnata*, and *Scutellaria baicalensis*), honey, and propolis [99]. There is no specific maximum concentration for the use of chrysin, but it is hypothesized that it should be kept low, based on the safety assessment of *S. baicalensis*-derived ingredients in cosmetics, which recommends a maximum concentration of 0.5% in leave-on cosmetic products [100]. Chrysin has poor water solubility (3.85 g/L) [100], which limits its bioavailability and use in aqueous formulations, being well dissolved in oils. Chrysin presents lower in vitro antioxidant properties [101] when compared with other flavones with a superior number of hydroxy groups, such as apigenin and luteolin. In in vivo models, its antioxidant and photoprotective activities are considered notable, due to its metal-chelating properties and ability to compete with the active site of the xanthine oxidase enzyme [102], and its ability to absorb UV radiation [103], respectively. Regarding its anti-inflammatory properties, chrysin has been reported for its ability to effectively inhibit the inducible form of nitric oxide synthase and the transcription factor NF-κB [101]. In vitro and in vivo studies have confirmed the anti-photoaging and anti-melanogenesis activities of chrysin, by respectively promoting the secretion of collagen type I by 121.54% at 25 µM and melanin content reduction by 20.35% at 25 µM, through the inhibition of melanogenic proteins and tyrosinase enzyme [104].

##### Resveratrol and Resveratrol Dimethyl Ether

**Figure 12:**
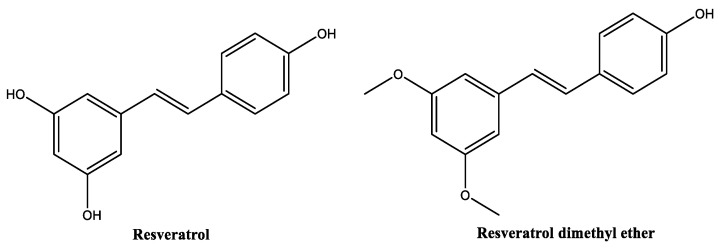
Chemical structure of resveratrol and resveratrol dimethyl ether.

Resveratrol (Figure 12), chemically known as 5-[(*E*)-2-(4-hydroxyphenyl)ethenyl]benzene-1,3-diol, is a naturally occurring polyphenol found in various plants, including grapes (*Vitis vinifera*), peanuts, and blueberries [105,106,107]. Resveratrol has been reported for its antitumor [108], antioxidant [105,109], anti-inflammatory [110], antimicrobial [111], anti-melanogenesis [112] and anti-aging [113,114] activities. It also plays a significant role in wound healing, and skin regeneration [115,116]. As resveratrol, resveratrol dimethyl ether (Figure 12) possesses antioxidant activity [117]. Resveratrol has been studied in concentrations ranging from 0.25% to 1% for cosmetic applications, which are generally considered safe and effective. However, according to the safety report for *Vitis vinifera* (grape)-derived ingredients, the maximum concentration could be as high as 3% [118]. Resveratrol possess oil-like properties (soluble in glycols and oils rich in triglyceride constituents). Its water solubility (30 mg/L) is the lowest when compared with the other phenolic compounds, and it is considered thermal- and photo-unstable, maintaining the stability at a pH range between 3 and 7 [118,119]. Resveratrol has already been investigated in in vitro and in vivo studies regarding its anti-melanogenic potential [112,120]. In the in vitro study, resveratrol demonstrated inhibitory effects on α-melanocyte stimulating hormone; however, its potential was inferior to resveratrol dimethyl ether [120]. Additionally, resveratrol dimethyl ether at a concentration of 10 µM inhibited the melanin content and intracellular tyrosinase activity by 63% and 58% [120], respectively. Moreover, the topical application of resveratrol has been demonstrated to significantly decrease hyperpigmentation on UVB-stimulated guinea pig skin [112]. Resveratrol has also demonstrated anti-senescent properties by modulating the senescence-associated secretory phenotype in THP-1 monocyte cells. This is achieved through a significant reduction in interleukins-1β and -6, microRNA-146a expression, and caspase-1 activation, alongside the upregulation of sirtuin-1 [121]. The senescence-associated secretory phenotype, which characterizes senescent cells, can contribute to the exacerbation of skin inflammation [121].

##### Glucosylrutin

**Figure 13:**
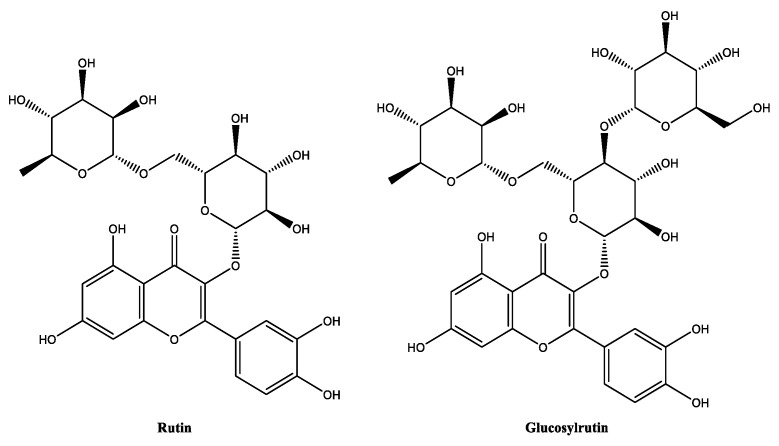
Chemical structure of rutin and glucosylrutin.

Glucosylrutin (Figure 13) is the glycosylated derivative of rutin, a naturally occurring flavonoid mainly found in citrus fruits [62,122] and which can be obtained through the enzymatic glycosylation of rutin [123]. α-glucosylrutin is an antioxidant compound, like rutin, but with improved water solubility and a higher epidermal bioavailability [62,124,125]. Glucosylrutin demonstrated a water solubility superior to its parent compound rutin, and helps to dissolves other ingredients, including resveratrol, in water-based cosmetic formulations [126]. It is stable in the range of skin pH, and possesses an improved photostability profile compared with rutin [124]. The excellent antioxidant potential of glucosylrutin has been shown in various experimental studies, both in vitro and in vivo, highlighting its potential as a UV absorber and skin-whitening agent [127]. Clinical studies have demonstrated that this flavonoid is also effective in the prevention of dermatologic diseases in which oxidative stress is of pathogenetic relevance, namely in polymorphous light eruption [124,125].

##### Isoquercitrin

**Figure 14:**
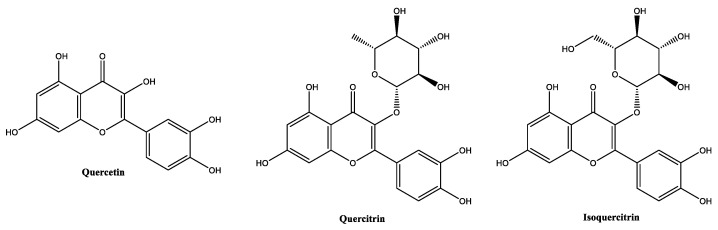
Chemical structure of quercetin, quercitrin, and isoquercitrin.

Isoquercitrin and quercitrin are both glycosides of quercetin (Figure 14), a natural flavonoid found in many plants, fruits and vegetables [9,83]. They differ in their sugar moieties attached at the C-3 position and, while quercitrin is classified as a flavonoid rhamnoside and is widely found in Loranthaceae sp. plants, isoquercitrin is a flavonoid glucoside and has been identified in Moraceae sp. plants [128]. Isoquercitrin is slightly soluble in water and is sensible to light, thus the packaging of the cosmetic products containing this flavonoid should be opaque in order to avoid possible light-induced degradation [129]. In terms of biological activity, isoquercitrin has exhibited higher ROS-scavenging activity and a higher metal-chelating effect than quercitrin, mainly due to the presence of an extra hydroxy group in the glycosylated moiety of isoquercitrin [128]. Even compared with quercetin (IC_50_ = 65.61 µg/mL), isoquercitrin (IC_50_ = 11.80 µg/mL) has demonstrated a superiority in antioxidant potential for the DPPH scavenging radical assay of almost six-fold, as well as stronger superoxide anion-scavenging activity (isoquercitrin: IC_50_ = 78.16 µM; quercetin: IC_50_ = 87.99 µM) [130]. The anti-inflammatory and anti-photoaging activities were also assessed in in vitro and in clinical studies. In vitro studies identified the potential of isoquercitrin as an anti-inflammatory agent, one with the ability to reduce the level of cyclooxygenase-2 and inflammatory cytokines, including interleukin-1β and -6 in UVB-irradiated epidermal skin cells [131], and by modulating the transforming growth factor-β/Smad pathway and metalloproteinase-1, contributing to collagen preservation, in UVA-exposed dermal skin cells [132]. A 28-day clinical study conducted with 30 volunteers aged between 31 and 55 years with the daily application of a formulation containing isoquercitrin at 0.5% was effective in the decrease of the facial trans-epidermal water loss and skin roughness, along with improved skin elasticity [132].

### 2.4. Structural Considerations

Among the various classes and subclasses of phenolic compounds, it is noteworthy that the most commonly found in the analyzed cosmetic products belong to the simple phenolics category—specifically, hydroxycinnamic acids and mono-/dihydroxy-phenols—and polyphenolics, particularly from the flavonoid and stilbene subclasses. It is possible to highlight some structural features, namely that all of the phenolic compounds possess at least one hydroxy group, mostly in *para*-position on the aromatic ring or in alternate positions, such as 1,3-dihydroxy, which is mainly correlated with the biosynthetic pathway [4]. Some methyl, *t*-butyl, and methoxy substitution groups are also present, in addition to the hydroxy groups. From the flavonoid derivatives, the presence of hydroxy groups in the *orto*- and *para*-positions in the A aromatic ring, and in consecutive positions in B aromatic ring, is a characteristic of their mixed biosynthetic pathway [6]. The A aromatic ring is formed via the acetate–malonate pathway, while the B aromatic ring is originated from the shikimate pathway. The chalcone scaffold is then synthesized by the action of chalcone synthase and, through cyclization, gives rise to flavonoids [6]. A total of two hydroxybenzoic and two hydroxycinnamic acids were detected in the categories of products analyzed. Hydroxybenzoic acids originate from shikimic acid, a precursor in the synthesis of aromatic amino acids, while hydroxycinnamic acids follow the phenylpropanoid pathway. Through the deamination of L-phenylalanine by the phenylalanine ammonia-lyase enzyme and successive hydroxylation reactions, hydroxycinnamic acids are formed [4].

The introduction of lipophilic substituent groups, such as aliphatic chains, could help to improve the lipophilic profile, allowing the compounds to present more of an affinity to an interaction with skin lipids and to skin permeation; nevertheless, the antioxidant potential of the compounds may decrease [133,134]. The glycosylation of the phenolic compounds is considered a chemical modification for improved stability and water solubility compared with the non-glycosylated compounds [135,136]. Long aliphatic chains have also been identified in some phenolic compounds, potentially enhancing their solubility in more hydrophobic-based formulations and improving their interaction with enzyme active sites [137]. This modification typically occurs through the esterification of phenolic acids or mono-/diphenols with fatty alcohols, contributing to the obtainment of compounds with affinities for both hydrophilic and lipophilic compounds [138].

The chemical structure of phenolic compounds enables them to absorb UV radiation, making them potential UV filters. Phenolic acids, such as hydroxybenzoic acids, have demonstrated the ability to absorb in the UVB range, while hydroxycinnamic acids can absorb both UVB and UVA radiation [139]. Three polyphenols (chrysin, isoquercitrin, and glucosylrutin) have already been reported for their UV-absorbing capacity [103,127,140,141].

Dermatokinetic parameters, including the ability to permeate skin layers, depend on physicochemical parameters such as lipophilic profile, molecular weight, solubility, and topological polar superficial area [142,143,144]. For instance, hydrophobic compounds with a smaller molecular weight could permeate to the dermis, while flavonoids with a higher molecular weight, and with a higher number of hydroxy groups, could have their permeation restricted to superficial skin layers. The same may apply to phenolic acids. Despite their small molecular weight, the lipophilic or hydrophilic nature of their substituent groups may influence their permeation into different skin layers [145].

Moreover, it is important to highlight the use of pure phenolic compounds versus natural extracts rich in phenolic compounds. Pure phenolic compounds enable accurate dosing and provide targeted effects, making them ideal for exploring specific mechanisms of action. However, the processes of extraction or chemical synthesis could be expensive and may overlook the combined effects noticed in natural extracts. Natural extracts containing phenolic compounds offer a diverse blend of bioactive components that may interact synergistically, potentially delivering greater health benefits compared with single, isolated compounds [146]; however, they could contribute to the overexploitation of natural resources and the loss of biodiversity, causing deforestation, and ecosystem degradation, as well as a decrease of species populations. More than 45% of a total of 444 sunscreen products (database collected in 2021) contained botanical extracts from *Glycine max*, *Vitellaria paradoxa*, *Persea gratissima*, *Glycyrrhiza inflata*, *Scutellaria baicalensis*, and *Aloe barbadensis* species, which have been reported to exhibit a synergistic effect due to the variety of phenolic compounds they contain [146]. A similar contribution of natural botanical extracts was observed in a work published by Ferreira et al., where they analyzed an anti-aging products database collected for the first time in 2011 and actualized in 2018 [147].

Table 2 presents a brief summary of the mechanism of action of the most used phenolic compounds in sun care and anti-aging cosmetic products.

## 3. Materials and Methods

### 3.1. Comprehensive Search of Literature for Introduction Section

The search of scientific literature undertaken for the introduction section was based on research and review articles found in the online databases PubMed, PubChem, and Scopus. This preliminary search was performed using the keywords “flavonoids” AND “skin care”, “skin aging”, “inflammation”, “oxidative stress”, “UV radiation”, and “oxidative damage of biomolecules”. This research was carried out from October 2024 to January 2025.

### 3.2. Data Collection

The label information of suncare products and anti-aging products from international cosmetic brands, marketed in Portuguese pharmacies, parapharmacies and online commerce was collected between 2021 and 2024 to assess the presence of phenolic compounds. Specific details related to the collection of the databases are listed below:

***Anti-aging****:* The label information of 771 anti-aging products, from 107 international cosmetic brands was collected and analyzed.

***Sunscreens****:* The label information of 444 sunscreens, from 43 international cosmetic brands, was collected and analyzed.

***Aftersun****:* The label information from 84 different aftersun products, from 41 international brands, was collected and analyzed.

### 3.3. Data Analysis

The phenolic compounds that are included in anti-aging and suncare products were listed according to the International Nomenclature of Cosmetic Ingredients (INCI). Phenols that belong to natural active secondary metabolites were considered. When their function did not fit this definition, they were grouped into a category called “Other phenolics”. The gathered data were examined observing the next parameters:

#### 3.3.1. Occurrence of Phenolic Compounds in Each Category of Cosmetic Products According to Chemical Classification and Origin

All ingredients were categorized according to chemical category into simple phenolics and polyphenols, following the criteria mentioned in the introduction section. The usage frequency (%) of all of the phenolic compounds was also determined according to their origin as natural and semi-synthetic/synthetic (semi-synt./synt.) ingredients.

#### 3.3.2. Usage Frequency of the Phenolic Compounds

The number of phenolic compounds presented in the label of each cosmetic product of each category was evaluated and their usage frequency was expressed as a percentage. The phenolic compounds presented in at least two categories of cosmetic products were identified and listed.

#### 3.3.3. Scientific Evidence Supporting the Effectiveness of the Phenolic Compounds to Prevent or Fight Solar-Induced Skin Damage

The scientific evidence for each ingredient was searched in the online databases ConsIng, Cosmetic Ingredient Review, the *Cosmetics & Toiletries* online magazine, INCI Decoder, COSMILE Europe, PubMed, PubChem, and Scopus. A broader search was performed using the keywords “INCI name” OR “synonyms”, when applicable, associated with the keywords: “skin aging”, “oxidative stress”, “inflammation”, “skin senescence”, “skin photodamage”, “sun damage”, “UV damage”, and “UV-induced damage”. This research was carried out from October 2024 to January 2025.

## 4. Strengths and Limitations of the Study

This study provides detailed insights into the active ingredients used in anti-aging, sunscreen, and aftersun products. It is based on careful analysis of label information and an extensive review of multiple scientific databases. The collected data were thoroughly organized and examined, offering a distinct and up-to-date perspective on the use of natural and synthetic/semi-synthetic phenolic compounds in the three categories of cosmetics designed to prevent or reduce skin aging caused by solar radiation. The research focused on the Portuguese market, with all of the products evaluated from international brands. Products were sourced from pharmacies, parapharmacies, and e-commerce platforms, potentially reflecting trends in the wider European market. This study has some limitations (geographical scope, data source, and distribution channels) which are listed below, as well as possible solutions.

- Examining data from other regions, especially non-European countries (e.g., North America, Asia, or South America), where consumer preferences, regulatory frameworks, and product formulations may differ significantly, in order to provide a more comprehensive global perspective.
- Exploring alternative distribution channels (supermarkets, or local specialized stores) would provide a broader view of market trends.
- Complementing label analysis with experimental studies or collaboration with manufacturers to access specific formulation data (concentration of the ingredients, and/or interactions between ingredients) could offer deeper insights into ingredient functionality.

## 5. Conclusions

**Figure 15:**
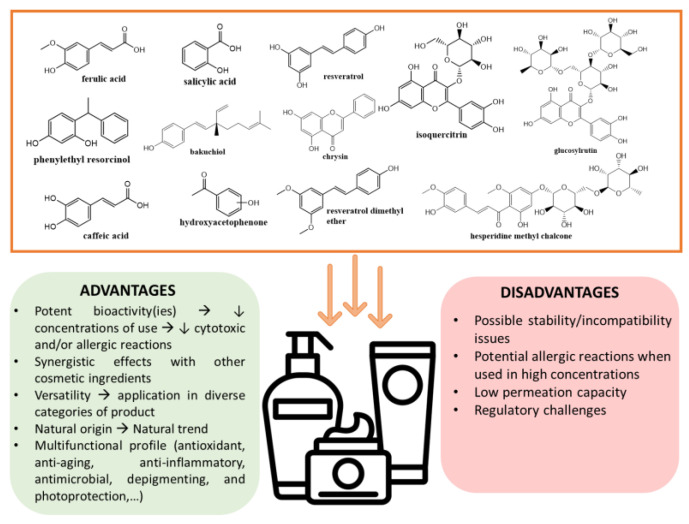
Most used phenolic compounds and their advantages and disadvantages when applied in cosmetic products.

A total of 1299 cosmetic products were analyzed, which contained a total of 28 phenolic compounds of natural and semi-synthetic/synthetic origin. Anti-aging products had the highest prevalence of phenolic compounds (13.2%), followed by sunscreen (5.2%) and aftersun products (4.8%). This distribution suggests that cosmetic industries recognize the potential of phenolic compounds, particularly in addressing age-related skin concerns and especially with regard to the thirteen compounds with proven effectiveness. Our analysis reveals that, for all of the phenolic compounds analyzed, antioxidant activity was the primary underlying mechanism of action of the phenolic compounds when fighting skin aging. The ability to scavenge ROS that are induced by extrinsic factors, particularly from solar radiation, helps to mitigate oxidative stress and maintain skin cell homeostasis. This prevents a cascade of reactions that activate biological biomarkers associated with skin aging, including the reduction of oxidative damage to biomolecules, attenuation of skin inflammation, modulation of melanin synthesis, and delay of cellular senescence. Certain phenolic compounds stand out for their multifunctionality and comprehensive scientific backing. The polyphenols resveratrol and chrysin have been demonstrated to have more comprehensive scientific studies supporting their diverse biological activities, while the simple phenolics bakuchiol and ferulic acid were the ingredients with more diversity regarding their mechanism of action. The photoprotective properties of ferulic acid and the caffeic acids, chrysin, and glucosylrutin highlight the potential of phenolics in sunscreen formulations. Additionally, the effectiveness of five phenolic compounds—salicylic acid, capryloyl salicylic acid, phenylethyl resorcinol, glucosylrutin, and isoquercitrin—is supported by clinical studies that validate their activity in at least one functional capacity. This type of study allows to corroborate the impact of phenolic compounds in cosmetic formulations and support their regulatory allegations, by bridging the gap between laboratory findings and practical applications. This trend towards evidence-based formulations may drive the increased incorporation of phenolic compounds in future skincare products (Figure 15).

Despite the extensive scientific evidence and claims supporting their efficacy and safety, the contribution of phenolic compounds in cosmetic products remains limited. This could be attributed to four main reasons: (i) the dominance of long-established ingredients with proven efficacy and safety profiles, which creates barriers to the adoption of new ingredients; (ii) intellectual property protections that restrict broader utilization; (iii) the challenges in formulating phenolic compounds with other cosmetic ingredients, including low solubility, stability issues, potential interactions with other ingredients, and limited skin permeability hindering their commercial use in formulas; and/or (iv) the preference for using natural extracts rich in phenolic compounds, following a trend of natural cosmetics, rather than incorporating individual phenolic compounds into formulations. In fact, the use of natural extracts rich in phenolic compounds has been found to be higher than the use of pure ingredients. In conclusion, despite the plethora of in vitro and in vivo studies supporting the effectiveness of phenolic compounds and their mechanisms of action for various skin care applications, their use in marketed cosmetic products, particularly in suncare and anti-aging formulations, remains relatively limited (Figure 15). Our findings suggest that the cosmetics industry is selectively taking up these ingredients, particularly in anti-aging formulations. However, there is room for growth in other categories, namely suncare products, given the broad spectrum of benefits these compounds offer. Future research should focus on overcoming formulation challenges and conducting more clinical studies to further validate the efficacy of phenolic compounds in various skincare applications.

## Figures and Tables

**Table 1:** Occurrence of phenolic compounds with a usage frequency above 1% or present in at least two categories of cosmetic products (anti-aging, sunscreen, and/or aftersun products).

Phenolic Compounds	Anti-Aging (n = 771)	Sunscreen (n = 444)	Aftersun (n = 84)
Hydroxyacetophenone	110 (14.3%)	5 (1.0%)	3 (3.6%)
Bakuchiol	24 (3.1%)	-	-
Capryloyl salicylic acid	22 (2.9%)	-	-
Salicylic acid	18 (2.3%)	-	3 (3.6%)
Ferulic acid	2 (0.3%)	9 (2.0%)	-
Caffeic acid	-	9 (2.0%)	-
Phenylethyl resorcinol	2 (0.3%)	2 (0.5%)	-
Hesperidin methyl chalcone	18 (2.3%)	-	-
Chrysin	12 (1.6%)	-	-
Resveratrol	9 (1.2%)	-	-
Resveratrol dimethyl ether	4 (0.5%)	4 (0.9%)	-
Glucosylrutin	1 (0.1%)	1 (0.2%)	-
Isoquercitrin	1 (0.1%)	1 (0.2%)	-

**Table 2:** Table of the mechanism of action of the most used phenolic compounds.

	Oxidative Stress	Inflammation	Oxidation of Biomolecules	Melanogenesis	Senescence	Photoprotective Properties	Skin Aging	Other Properties
**Hydroxyacetophenone** **(anti-aging, sunscreen, and aftersun products)**	✓ Scavenges ROS	✓ Modulates COX-2 and reduces skin irritation	✓ Neutralizes lipidic radicals	-	-	-	-	Fragrances, and preservative
**Bakuchiol** **(anti-aging products)**	✓ Neutralizes ROS and donates hydrogen atoms	✓ ↓ IL-6, and PGE2, inhibits expression of the iNOS	-	✓ Blocks α-MSH and activates tyrosinase	✓ Stimulates production of growth factors	-	✓ Reduces fine lines and wrinkles, and promotes collagen production	Antimicrobial, skin conditioning, and emollient
Salicylic acid * **(anti-aging and aftersun products)**	✓ Scavenges ROS	✓ ↓ Skin irritation	-	✓ Inhibits of melanosome transport	-	-	-	Fragrance, preservative, skin conditioning, keratolytic
Capryloyl salicylic acid * **(anti-aging products)**	✓ Scavenges ROS	✓ ↓ Skin redness and oedema	-	✓ ↓ Dark brown spots	-	-	✓ ↓ Skin wrinkles	Skin conditioning
**Ferulic acid** **(anti-aging and sunscreen products)**	✓ Forms stable phenoxy radical and participates in hydrogen atom transfer	✓ Downregulates MAPK pathway	✓ Protects DNA from oxidative damage	✓ ↓ Tyrosinase activity, interacts with α-MSH, and inhibits CK2	-	✓ Boosts UV radiation absorption when in combination with UV filters and protects against UVB radiation	✓ ↑ Skin appearance, firmness and elasticity, activates collagen and elastin production	Antimicrobial
**Caffeic acid** **(sunscreen products)**	✓ Establishes intramolecular hydrogen bonds and donates hydrogen atoms	✓ ↓ IL-1β and -6 levels	-	✓ Inhibits casein kinase 2 and α-MSH	-	✓ Protect against UVB radiation	✓ Inhibits MMP-1, MMP-2, and MMP-8	Fragrance
Phenylethyl resorcinol * **(anti-aging and sunscreen products)**	✓ Scavenges ROS	-	-	✓ Activates p44/42 MAPK pathway and acts as skin lightening agent	-	-	-	-
**Hesperidin methyl chalcone** **(anti-aging products)**	✓ Scavenges ROS, possesses quenching properties, and metal chelating effects	✓ ↓ IL-1β, -6, and -10 levels	✓ Inhibits lipid peroxyl radicals	-	-	-	✓ Inhibits MMP-1 and MMP-8	-
**Chrysin** **(anti-aging products)**	✓ Metal-chelating properties and interacts with XO enzyme	✓ Inhibits the iNOS and NF-κB	-	✓ Inhibits of melanogenic proteins and tyrosinase enzyme	-	✓ Absorbs UVA and UVB radiation	✓ Promotes the secretion of collagen type I	Skin conditioning
**Resveratrol** **(anti-aging products)**	✓ Scavenges ROS and donates hydrogens	✓ ↓ IL-1β, and -6 levels		✓ Inhibits α-MSH, and reduces the expression of tyrosinase-related proteins 1 and 2	✓ Modulates SASP, decreases the microRNA-146a expression levels and downregulates caspase-1		✓ Inhibits MMP-1, MMP-2, MMP-7 and MMP-9	Skin protecting
**Resveratrol dimethyl ether** **Anti-aging and sunscreen products)**	✓ Scavenges ROS scavenger and donates hydrogens	-	-	✓ Inhibits α-MSH and tyrosinase activity	-	-		Skin conditioning
Glucosylrutin * **(anti-aging and sunscreen products)**	✓ Scavenges ROS	-	-	✓ Skin whitening agent	-	✓ Ability to absorb UV radiation in combination with cinnamic acids	-	-
Isoquercitrin * **(anti-aging and sunscreen products)**	✓ Scavenges ROS scavenging activity and has metal chelating properties	✓ Inhibits COX-2, reduces levels of IL-1β and -6, and modulates TGF-β/Smad pathway	-	-	-	-	✓ Inhibits MMP-1, and reduces skin roughness	-

* ingredients that demonstrated activity in clinical studies for at least one activity; ↓; decrease; ↑: increase; α-MSH: alpha-melanocyte stimulating hormone; COX-2: cyclooxygenase-2; CK2: casein kinase 2; ILs: interleukins; iNOS: inducible form of nitric oxide synthase; MAPK: mitogen-activated protein kinase; MMP: metalloproteinase; NF-κB: nuclear factor-κB; PGE2; prostaglandins E2; ROS: reactive oxygen species, SASP: senescence-associated secretory phenotype; TGF-β: transforming growth factor-beta; XO: xanthine oxygenase.

## Data Availability

Data are contained within the article and its Supplementary Materials.

## Supplementary Materials

The following supporting information can be downloaded at https://www.mdpi.com/article/10.3390/molecules30071423/s1, Table S1: CVS document containing the list of the phenolic compounds found in each category of cosmetic products (sunscreen, aftersun, and anti-aging products).
